# Dose–response relationships of normal blood lipid levels in metabolic and endocrine diseases: mechanistic similarities, differences, and functional insights

**DOI:** 10.3389/fendo.2026.1768784

**Published:** 2026-04-16

**Authors:** Xinyue Hu, Xiaofang Yang, Zhou Zhu, Zhihong Yang, Pengyu Wang, Min Wu, Ning Zhang

**Affiliations:** 1 School of Acupuncture and Tuina, Guizhou University of Traditional Chinese Medicine, Guiyang, Guizhou, China; 2 The First Affiliated Hospital, Guizhou University of Traditional Chinese Medicine, Guiyang, China

**Keywords:** dose–response relationship, endocrine diseases, mechanisms, metabolic diseases, normal blood lipids, risk assessment, similarities and differences

## Abstract

As major products of lipid metabolism, blood lipids not only participate in the maintenance of energy homeostasis and the formation of cellular structures, but are also closely involved in endocrine signaling regulation. Although hyperlipidemia is a well-recognized pathogenic factor, a systematic understanding of the potential effects of lipid fluctuations within the normal reference range on metabolic and endocrine homeostasis remains lacking. Current epidemiological evidence suggests that the relationship between blood lipid levels and health risk does not follow a single all-or-none threshold pattern. Even within the clinically defined normal range, variations in certain lipid components may still show dose–response relationships with disease risk, and this continuous effect appears to be complex and heterogeneous across different metabolic and endocrine disorders. This review aims to systematically summarize the available evidence regarding the associations between different lipid components within the normal range and the risk of major metabolic and endocrine diseases. Particular emphasis is placed on comparing the similarities and differences in dose–response relationships across disease spectra and on exploring their potential shared and disease-specific mechanisms, including lipotoxicity-mediated β-cell dysfunction, the early initiation of insulin resistance, and abnormalities in feedback regulation along endocrine axes. Overall, traditional static lipid reference values may not always adequately reflect an individual’s true metabolic risk. Future research should move beyond the conventional concept of achieving lipid targets and shift toward more refined risk assessment based on dose–response relationships, with the aim of clarifying the risk gradients of different lipid components within the normal lipid range and the contexts in which they apply. Such efforts may provide a basis for the early identification of metabolic and endocrine diseases, lifestyle intervention, and individualized risk management.

## 1 Introduction

Metabolic and endocrine homeostasis constitutes an essential foundation for the maintenance of normal physiological function (1). Disruption of this regulatory balance drives the onset and progression of complex pathological processes, including metabolic dysfunction-associated steatotic liver disease (MAFLD), diabetes mellitus (DM), obesity (OB), and thyroid disease (TD), thereby posing a major global public health challenge (2, 3). As key substances involved in the maintenance of cellular structure and the regulation of energy metabolism, blood lipids have long been recognized as closely associated with the risk of cardiovascular and metabolic diseases. Typical pathological alterations, including elevated low-density lipoprotein cholesterol (LDL-C), triglycerides (TG), and total cholesterol (TC), together with reduced high-density lipoprotein cholesterol (HDL-C), are well established as important risk-related features (4–7). However, whether the clinically defined “normal” lipid range is truly equivalent to an optimal physiological state is increasingly being questioned. Accumulating evidence suggests that even subtle fluctuations in lipid profiles within the reference range may still influence long-term metabolic risk by inducing early pathological processes such as atherosclerosis (AS), insulin resistance (IR), and chronic inflammation (8, 9). These observations indicate that the traditional binary distinction between “health” and “disease” may overlook the continuous nature of dose–response risk patterns, the underlying mechanisms of which remain to be further elucidated.

Accordingly, this review aims to systematically examine the evidence regarding the associations between lipid variation within the “normal” range and the metabolic and endocrine diseases discussed above. By comparing the shared and distinct features of the dose–response relationships among different lipid components and exploring their potential molecular basis, it further seeks to clarify how subtle lipid fluctuations may influence metabolic homeostasis and to provide a theoretical basis for more refined and forward-looking lipid risk assessment and management.

## 2 Dose–response relationships between normal blood lipid levels and metabolic–endocrine diseases

### 2.1 Classification of blood lipid components and their normal-range fluctuations

#### 2.1.1 Dual roles of major blood lipid components

Major blood lipid components exhibit distinct dual roles in maintaining physiological homeostasis and mediating pathological injury. Under physiological conditions, HDL-C exerts vasoprotective effects through reverse cholesterol transport and anti-inflammatory mechanisms. However, under pathological states such as chronic inflammation or DM, HDL-C particles are prone to structural remodeling and functional impairment, potentially adopting a pro-inflammatory phenotype and thereby losing their protective capacity (10, 11). LDL-C, the primary carrier delivering cholesterol to peripheral tissues, is essential for sustaining cell membrane integrity and steroid hormone synthesis. Nevertheless, its pathological accumulation and subsequent oxidative modification constitute key mechanisms initiating vascular endothelial inflammation, foam cell formation, and the onset of AS (12). TG serve as a primary form of energy storage in the body and as an essential source of fatty acids(FAs). However, hypertriglyceridemia is frequently accompanied by IR, which not only constitutes a central feature of metabolic syndrome and elevates the risk of atherosclerotic cardiovascular disease, when markedly elevated, is also associated with the onset of acute pancreatitis (13–16). Moreover, TC as a regulator of cellular membrane fluidity and a precursor of bioactive molecules, is another critical factor; its homeostatic imbalance represents a significant risk factor driving the development of cardiovascular disease (17, 18) (**Table 1**).

**Table 1:** Biological characteristics of major blood lipid components and their dual clinical effects.

Lipid type	Biosynthetic pathway	Beneficial factors	Adverse factors
HDL-C	Synthesized by the liver and small intestine; apoA-I forms nascent HDL with the ABCA1 transporter; matures under the action of LCAT; completes reverse cholesterol transport via SR-BI or CETP (19).	①RCT: Removes cholesterol from peripheral tissues and reduces foam cell formation;②Anti-inflammatory: Suppresses adhesion molecules and inflammatory cytokines; ③Antioxidative: Carries PON1 to degrade oxidized lipids;④Endothelial protection: Promotes NO production and improves vasodilation;⑤Antithrombotic: Modulates platelet function (20).	① Dysfunction: In states such as DM, chronic inflammation, and kidney disease, HDL loses its anti-inflammatory and antioxidative capacities and may even become pro-inflammatory; ② Extremely high HDL-C levels (men>90mg/dL, women>100–110mg/dL) exhibit a U-shaped association with increased all-cause mortality;③ Pharmacological elevation of HDL-C (e.g., CETP inhibitors, niacin) has not yielded cardiovascular benefits (21, 22).
LDL-C	VLDL, synthesized and secreted by the liver, are gradually hydrolyzed in circulation by lipoprotein lipase and hepatic lipase, first converting to IDL and eventually forming LDL. Mature LDL particles enter cells via LDL receptors to supply cholesterol for metabolic use (23).	①Provides cholesterol for cell membranes, maintaining structure and function; ②Serves as a precursor for steroid hormone and bile acid synthesis, supporting endocrine and digestive functions; ③Participates in immune defense by binding to and neutralizing bacterial toxins (24).	①Elevated LDL-C deposits in the vascular wall and oxidizes to ox-LDL, triggering endothelial dysfunction and inflammatory responses, thereby promoting foam cell formation and AS plaque development; ②Facilitates vasoconstriction and thrombosis, increasing ASCVD risk; ③LDLR defects, as seen in familial hypercholesterolemia, markedly elevate the risk of premature cardiovascular disease (25, 26).
TG	①Exogenous pathway: Dietary fats are hydrolyzed by pancreatic lipase in the small intestine into FAs and glycerol, re-esterified into TG, and packaged into chylomicrons entering the circulation; ②Endogenous pathway: The liver synthesizes TG from glucose and free FAs, secretes them into VLDL, and transports them to peripheral tissues (27).	①Serves as the main energy reserve of the body, providing FAs and glycerol during increased energy demand; ②TG in adipose tissue helps maintain thermal stability and buffers vital organs; ③TG in breast milk provides essential energy and FAs for infant growth (28, 29).	①Hypertriglyceridemia is frequently associated with OB, IR, and metabolic syndrome, promoting atherosclerosis; ②Extremely high TG levels (>1000 mg/dL) constitute a major risk factor for acute pancreatitis; ③TG-rich lipoprotein remnants increase ASCVD risk (28, 30, 31).
TC	①Exogenous pathway: Dietary cholesterol is absorbed through the intestine into the circulation; ②Endogenous pathway: Cholesterol is synthesized by the liver and small intestine (32).	①Serves as a fundamental component of cell membranes, regulating membrane fluidity and signal transduction; ②Acts as a precursor for steroid hormones, bile acids, and vitamin D, maintaining endocrine, digestive, and calcium homeostasis; ③Promotes myelin formation and neural conduction in the nervous system (33, 34).	①Elevated TC levels (particularly with a high proportion of LDL-C) increase AS risk, promote plaque formation, and elevate the risk of myocardial infarction and stroke; ②Familial hypercholesterolemia leads to persistently elevated TC, markedly increasing the risk of premature ASCVD; ③Persistently high TC levels serve as an important indicator for cardiovascular event risk stratification (35–37).

HDL-C, HDL cholesterol; LDL-C, LDL cholesterol; TG, Triglyceride; TC, Total cholesterol; VLDL, Very-low-density lipoproteins; IDL, intermediate-density lipoproteins; RCT, Randomized Controlled Trial; FAs, Fatty acids; AS, Atherosclerosis; ASCVD, Atherosclerotic Cardiovascular Disease; IR, Insulin Resistance; OB, Obesity.

#### 2.1.2 Dynamics and influencing factors of normal ranges

**Figure 1:**
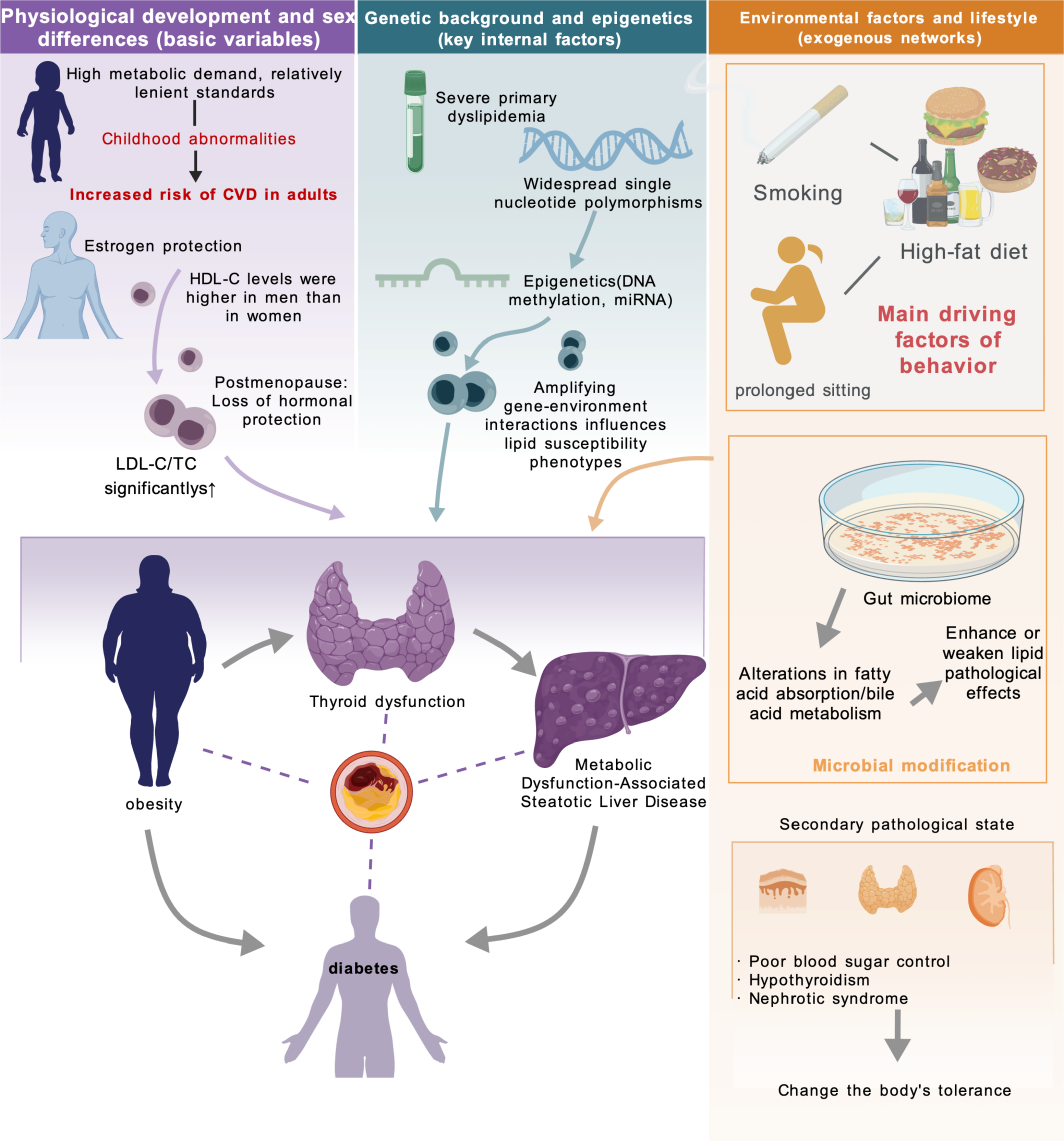
Factors affecting the normal range of blood lipids.

The “normal range” of blood lipids represents a dynamic interval, and its clinical interpretation should be individualized (38). This dynamic nature arises because the dose–response relationship between blood lipid levels and the risk of metabolic and endocrine diseases does not follow a simple linear pattern; rather, it is profoundly influenced by individual physiological characteristics, genetic background, and environmental factors, thereby exhibiting substantial heterogeneity, as illustrated in **Figure 1** (39).

Physiological development and sex differences represent fundamental determinants for establishing blood lipid reference standards (40, 41). Lipid assessment in children and adolescents typically relies on age-specific reference standards rather than the direct application of adult criteria (42, 43). For example, the generally acceptable level of LDL-C in children and adolescents is <110 mg/dL, whereas the optimal LDL-C level for healthy adults is typically defined as <100 mg/dL (44). Moreover, epidemiological studies have demonstrated that dyslipidemia during childhood is associated with an increased risk of cardiovascular disease in adulthood, suggesting that the pathological cumulative burden of cardiovascular risk may originate early in life (45). In terms of sex differences, HDL-C levels in premenopausal women are typically higher than those observed in men. Accordingly, commonly used threshold values for low HDL-C<50 mg/dL in women and <40 mg/dL in men (46, 47). However, during the perimenopausal and menopausal transition, the decline in estrogen levels, together with age-related physiological changes, is often accompanied by increases in TC and LDL-C in women; consequently, cardiovascular risk correspondingly increases (48, 49). Specific blood lipid reference ranges stratified by age and sex are summarized in **Table 2** (50–52).

**Table 2:** Reference ranges for blood lipids by age and sex (mg/dL).

Lipid type	Age		Sex	Normal level	Borderline high	High	Very high
TC	Children and adolescents		female	<170	170–199	≥200	not applicable
			male	<170	170–199	≥200	not applicable
	Adults		female	<200	200-239	≥240	not applicable
			male	<200	200-239	≥240	not applicable
LDL-C	Children and adolescents		female	<110	110-129	≥130	not applicable
			male	<110	110-129	≥130	not applicable
	Adults		female	<100	130-159	160-189	≥190
			male	<100	130-159	160-189	≥190
HDL-C	Children and adolescents		female	≥50	40-49	<40	not applicable
			male	≥50	40-49	<40	not applicable
	Adults		female	≥60	50-59	<50	not applicable
			male	≥60	40-59	<40	not applicable
TG	Children and adolescents	2–9 years old	female	<75	75-99	≥100	not applicable
			male	<75	75-99	≥100	not applicable
		10–19 years old	female	<90	90-129	≥130	not applicable
			male	<90	90-129	≥130	not applicable
	Adults		female	<150	150-199	200-499	≥500
			male	<150	150-199	200-499	≥500

Genetic background and epigenetic mechanisms constitute key intrinsic determinants of inter-individual variability in sensitivity to blood lipid dose–response relationships (53, 54). Apart from a limited number of high-effect genetic mutations that cause severe primary dyslipidemia, widely distributed single-nucleotide polymorphisms may also collectively influence lipid traits within a polygenic framework and thereby increase an individual’s genetic susceptibility to dyslipidemia (55). In addition, DNA methylation, an epigenetic modification that does not alter the DNA sequence, can participate in the regulation of lipid-related gene expression and has been associated with lipid traits and dyslipidemia (56).

Environmental factors and lifestyle behaviors influence lipid levels and contribute to the occurrence and progression of dyslipidemia. Unhealthy dietary patterns, physical inactivity, and smoking are closely associated with abnormal lipid profiles and represent important modifiable behavioral risk factors (57–59). Emerging evidence further suggests that the gut microbiome participates in this process, as intestinal microbial communities can regulate host lipid metabolism and lipid homeostasis by influencing lipid absorption, bile acid metabolism, and related metabolic signaling pathways (60, 61). In addition, secondary pathological conditions, including poor glycemic control, hypothyroidism, and nephrotic syndrome, may also lead to or exacerbate dyslipidemia (58, 62).

In summary, genetic, physiological, and environmental factors all influence lipid levels and their clinical significance. This indicates that the normal range of blood lipids should be regarded primarily as a reference rather than a uniform risk threshold applicable to all individuals. Even when lipid levels fall within the general reference range, the associated disease risk may still vary among individuals. Therefore, clinical assessment should not rely solely on a single numerical cutoff value but should instead incorporate a comprehensive evaluation that considers age, sex, genetic background, comorbid conditions, and lifestyle factors.

### 2.2 Common theoretical models of dose–response relationships and their application in studies of normal blood lipid levels

Dose–response relationships constitute a fundamental basis for epidemiological risk assessment and causal inference (63). In studies examining the association between blood lipids and health outcomes, linear models have revealed a continuous proportional relationship between exposure levels and risk. Existing evidence suggests that risk variation in certain lipid related indicators does not necessarily occur only after exceeding traditional abnormal thresholds; rather, differences in risk may also exist across varying levels within lower or reference ranges. Even within the conventionally defined normal range or the upper normal interval, relatively higher TG levels have been associated with an increased risk of cardiovascular events (64). In addition, elevations in TG related indicators, such as the triglyceride glucose index (TyG), have been linked to a higher risk of MAFLD (65). These findings indicate that evaluating lipid related risk solely on the basis of traditional normal or abnormal thresholds may be insufficient. Instead, attention should also be directed toward the differences in risk associated with varying levels within the reference range.

In contrast to a unidirectional increasing relationship, J shaped or U shaped curves reveal the bidirectional nature of risk, suggesting that certain lipid indicators may be associated with an increased risk of adverse outcomes when levels are either excessively low or excessively high. HDL-C represents a common example of a U shaped association, as its relationship with cardiovascular events and all-cause mortality can exhibit a nonlinear pattern (66). In addition, LDL-C has been reported to show a J shaped association with all-cause mortality and with certain cardiovascular mortality outcomes (67). This pattern suggests that the clinically defined “normal range” does not necessarily correspond to the “lowest-risk interval,” thereby emphasizing the importance of identifying an “optimal physiological window” within the normal range to minimize potential heterogeneity in risk.

In addition, composite lipid indicators may also exhibit U shaped nonlinear associations with disease risk. A cross sectional study conducted in an elderly population reported a significant U shaped relationship between the TC/HDL-C and the risk of MAFLD, with an inflection point at 3.56 (68). The lower and higher ranges may correspond to distinct metabolic states: the lower range may be related to age associated vulnerability conditions such as malnutrition or chronic inflammation, whereas the higher range is more likely associated with atherogenic dyslipidemia and IR (69, 70). These findings suggest that, in elderly populations, assessing metabolic risk solely on the basis of traditional reference ranges may be insufficient, and the optimal reference interval for the TC/HDL-C ratio requires further investigation.

Threshold models define critical points at which risk undergoes a marked change. In addition to clinically recognized cutoff values, evidence suggests that earlier points of risk transition may exist even within the conventionally defined normal range. For example, increases in TG levels, even when remaining within the normal range, have been associated with a higher risk of IR and abnormalities in glucose metabolism (71, 72). Moreover, elevations in TG related indicators, such as the TyG and the TG/HDL-C, have been linked to early vascular alterations, including arterial stiffness (73). Identifying these earlier signals of risk transition may facilitate more refined risk stratification and provide a basis for early detection and individualized intervention.

### 2.3 Associations between metabolic–endocrine diseases and lipid metabolism

Metabolic and endocrine disorders generally have a complex etiological basis, and their development and progression are typically the result of the combined influence of genetic background, environmental exposures, lifestyle factors, and multiple metabolic pathways. Under the influence of these factors, dysregulation of lipid metabolism is considered a key component. It is closely associated with disease occurrence and progression not only when lipid levels are markedly abnormal, but also when lipid levels fall within the conventionally defined normal range, where metabolic alterations related to increased risk may already be present.

#### 2.3.1 Type 2 diabetes

DM comprises a heterogeneous group of metabolic disorders, among which type 2 diabetes mellitus (T2DM) is the most common clinical subtype. T2DM is characterized by chronic hyperglycemia, with its pathological basis primarily involving IR and progressive β-cell secretory dysfunction (74). IR often precedes detectable hyperglycemia and constitutes a pivotal process driving the transition from compensatory to decompensated disease stages. As pancreatic β-cells are subjected to prolonged secretory demand, their functional capacity gradually declines, ultimately resulting in impaired glycemic regulation (75).

Dysregulated lipid metabolism plays a central role in both the pathogenesis and progression of T2DM. Lipotoxicity represents a major mechanism: excessive accumulation of free fatty acids (FFAs) and TG can impair insulin signaling in hepatocytes and skeletal muscle cells, while simultaneously inducing oxidative stress, endoplasmic reticulum (ER) stress, and inflammatory responses in pancreatic β-cells, thereby further compromising their function (76). Adipose tissue dysfunction also contributes to IR; hypertrophic adipocytes trigger chronic low-grade inflammation and adipokine imbalance, systemically reducing insulin sensitivity. Moreover, ectopic fat deposition in non-adipose tissues, including the liver, muscle, and pancreas, disrupts local metabolic homeostasis and promotes IR, serving as a critical mechanistic link between OB and T2DM (77).

Even when certain lipid related indicators fall within the traditional reference range, variations in their levels may still be associated with metabolic risk related to T2DM (78). Evidence indicates that, even in individuals with normal glucose tolerance, an attenuated decline in FFA following a glucose load is associated with an increased future risk of T2DM (78). In addition, elevated fasting FFA levels have been linked to reduced insulin secretion and are associated with a higher risk of subsequently developing impaired glucose tolerance or T2DM (79). TG/HDL-C is closely related to IR and may be used to identify or predict the risk of metabolic syndrome (80). Elevations in FFA, TG and the TG/HDL-C ratio may therefore be regarded as early metabolic signals of IR and an increased future risk of T2DM. Accordingly, assessing T2DM risk solely on the basis of whether traditional lipid targets are achieved may be insufficient; attention should also be directed toward variations and dynamic characteristics of lipid related metabolic indicators within the normal range.

#### 2.3.2 Obesity

OB represents a common underlying factor for multiple metabolic and endocrine diseases, with its hallmark being dual abnormalities in adipose tissue quantity and function. During OB, adipose tissue undergoes not only volumetric and numerical expansion but also profound dysregulation of lipid metabolism, encompassing enhanced lipolysis, imbalanced fatty acid re-esterification, aberrant lipid droplet dynamics, and altered adipokine secretion. Consequently, adipose tissue transitions from a primary energy storage depot to a source of chronic inflammation and metabolic stress, thereby impairing the responsiveness of liver, muscle, and cardiovascular tissues to lipid-mediated signaling. Hence, OB not only induces lipid metabolic disturbances but also modulates the physiological impact of “normal” blood lipid levels, such that identical lipid concentrations may elicit divergent metabolic consequences depending on the OB state.

In this context, metabolically healthy obesity (MHO) and metabolically unhealthy obesity (MUO) have garnered considerable attention. Although individuals in both categories may present with similar body mass index (BMI) or overall fat mass, their metabolic profiles differ substantially. MHO is characterized by more favorable fat distribution, lower levels of adipose tissue inflammation, and more stable adipokine secretion, thereby preserving normal responsiveness to insulin and lipid signaling. In contrast, MUO is typified by visceral fat accumulation, adipocyte hypertrophy and dysfunction, chronic inflammatory activation, and pronounced ectopic fat deposition (81). Certain lipid related indicators may help distinguish MHO from MUO. Existing evidence suggests that higher TG levels and lower HDL-C levels are associated with an increased risk of transition from MHO to MUO. In addition, individuals with MUO often exhibit more pronounced features of atherogenic dyslipidemia, such as an increased proportion of small dense LDL particles (82, 83). These alterations suggest that abnormalities in lipid metabolism may already emerge at relatively early stages of metabolic deterioration.

Therefore, OB should be regarded not as a singular condition but as a syndrome characterized by substantial metabolic heterogeneity. Comprehensive evaluation of lipid phenotypes in obese individuals—particularly with attention to “subclinical variations within the normal range”—can facilitate earlier detection of metabolic risk, deepen insights into the continuum linking lipid metabolism and disease development, and provide critical evidence to guide precision intervention strategies.

#### 2.3.3 Metabolic dysfunction-associated steatotic liver disease

MAFLD (the term MAFLD is used throughout this review, although some of the original studies discussed in later sections adopted the term NAFLD) is a spectrum of disorders characterized by abnormal lipid accumulation in hepatocytes, ranging from simple steatosis to nonalcoholic steatohepatitis (NASH), and may further progress to liver fibrosis, cirrhosis, or even hepatocellular carcinoma (84). With the continuous global increase in the prevalence of OB and T2DM, MAFLD has become one of the most common chronic liver diseases and is closely associated with metabolic dysfunction (85, 86). Importantly, its pathogenesis extends beyond localized hepatic lesions and reflects systemic metabolic dysregulation.

Dysregulated lipid metabolism constitutes the central pathological basis of MAFLD. Under physiological conditions, the liver maintains lipid homeostasis through coordinated processes including fatty acid uptake, *de novo* lipogenesis (DNL), β-oxidation of FAs, and very-low-density lipoprotein (VLDL) secretion. In MAFLD patients, however, increased peripheral FFA delivery, overactive DNL, impaired fatty acid oxidation, and reduced VLDL export collectively lead to hepatic accumulation of TG and cholesterol esters. This lipid overload induces steatosis, oxidative stress, inflammation, and hepatocellular injury, thereby promoting disease progression toward more advanced stages (87).

IR and MAFLD exhibit a mutually reinforcing relationship. Systemic IR can promote dysregulated lipolysis and increase the flux of FFA to the liver. In contrast, hepatic IR is associated with enhanced DNL and abnormalities in glucose metabolism, which further aggravate hepatic lipid accumulation (88). Furthermore, dysregulation of the gut–liver axis modulates lipid metabolism and promotes inflammation and fibrosis via short-chain FAs, endotoxins, and bile acids (89). However, lipid abnormalities in MAFLD are not fully captured by conventional blood lipid indices. Existing studies indicate that TG, HDL-C, LDL-C and their related ratios are associated with the risk of MAFLD (90). However, these indicators primarily reflect lipid status in the peripheral circulation and cannot directly substitute for the assessment of hepatic lipid accumulation. Additional evidence suggests that an elevated TG to HDL-C ratio is associated with a higher prevalence of fatty liver, and this relationship may exhibit a nonlinear pattern (91). These findings indicate that assessing MAFLD risk solely on the basis of traditional lipid thresholds may be insufficient.

Therefore, comprehensive risk assessment in MAFLD necessitates consideration of fine-scale alterations in lipid profiles and related metabolic markers, enabling earlier and more precise detection and intervention.

#### 2.3.4 Thyroid diseases

Thyroid dysfunction represents a critical regulator of energy metabolism and lipid homeostasis. Thyroid hormones maintain lipid metabolic equilibrium by modulating cholesterol synthesis, bile acid metabolism, fatty acid oxidation, and the production and clearance of various lipoproteins. Specifically, they regulate HMG-CoA reductase activity, upregulate low-density lipoprotein receptor (LDLR) expression to enhance LDL-C clearance, and modulate the activities of lipoprotein lipase and hepatic lipase. Consequently, even subtle alterations in thyroid function can exert significant effects on lipid metabolism.

Hypothyroidism frequently results in increased cholesterol synthesis, reduced LDL clearance, and abnormal lipoprotein remodeling, leading to elevated levels of LDL-C, TC, and potentially atherogenic lipoproteins (92). In contrast, hyperthyroidism accelerates cholesterol catabolism and enhances lipoprotein clearance, thereby manifesting as reduced blood lipid levels (93). Notably, clinically relevant lipid alterations may already emerge at subclinical or subtle stages rather than only in overt thyroid dysfunction. Even minor variations in thyroid-stimulating hormone within the conventional reference range have been associated with modest changes in LDL-C and TG, while free thyroxine (T4) levels within the nomal range may also be related to lipid variation, particularly TG-related changes (94–96). Although such subtle changes may be overlooked in routine lipid assessments, they can nonetheless influence overall metabolic homeostasis. Subclinical hypothyroidism is closely associated with the progression of AS, endothelial dysfunction, aggravated IR, and increased risk of metabolic syndrome (97–99). Furthermore, hepatic lipid metabolism is highly sensitive to thyroid hormones, which can amplify or attenuate the impact of other metabolic disorders on blood lipid profiles (100, 101).

Although multiple metabolic diseases exhibit lipid dysregulation, their underlying pathophysiological mechanisms are not entirely identical. Variations exist in fatty acid supply, lipid-handling capacity, lipoprotein regulation, inflammatory profiles, and tissue-specific lipid deposition. Consequently, identical blood lipid levels may carry distinct physiological and pathological significance depending on the specific disease context.

## 3 Common mechanistic pathways underlying dose–response relationships of normal blood lipid levels

Accumulating evidence indicates that even subtle fluctuations in blood lipids within the conventional normal range can exert measurable effects on metabolic homeostasis. Lipid components within this range are capable of regulating multiple key metabolic processes in a dose-dependent manner, thereby influencing susceptibility to metabolic and endocrine diseases, modulating disease progression, and shaping clinical phenotypes. These dose–response relationships primarily arise from three shared mechanisms: common physiological and pathological foundations, reliance on overlapping molecular signaling pathways, and induction of analogous effects at the cellular level. It is precisely these shared characteristics that allow mild variations in lipid levels within the normal range to influence the overall metabolic milieu.

### 3.1 Common physiological and pathological basis

**Figure 2:**
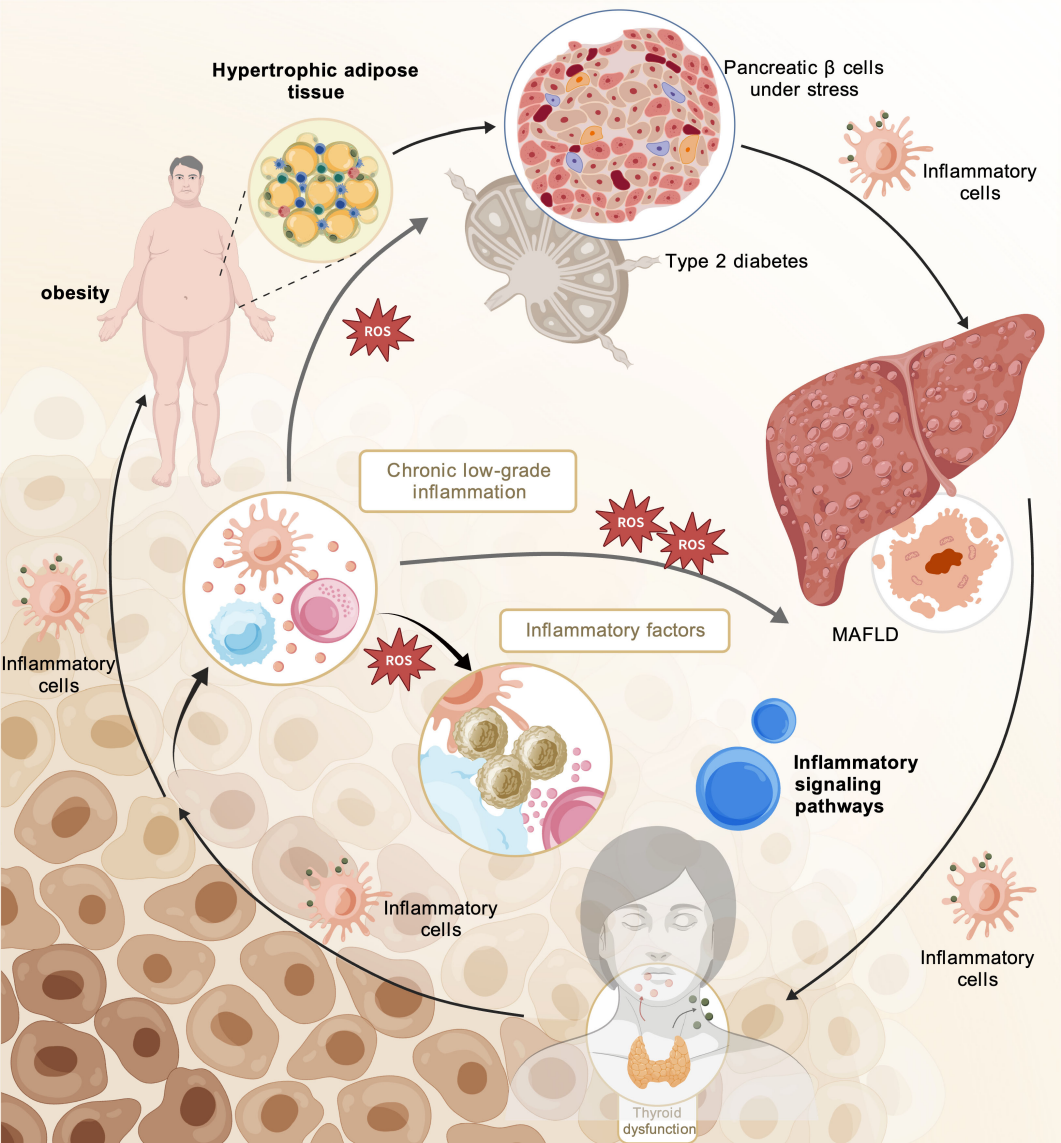
Common physiological and pathological basis.

Chronic low-grade inflammation represents a common feature of metabolic diseases, and dyslipidemia may participate in this process (102). Existing evidence suggests that, even within the conventionally defined normal range, relatively higher TG levels are associated with IR and with an increased future risk of T2DM (71, 72). In addition, elevations in TG related indicators, such as TyG and the TG to TG/HDL-C, have also been associated with inflammatory status and with increased cardiometabolic risk (103, 104). These findings suggest that variations in lipid levels within the normal range do not necessarily indicate uniform risk, and that in some individuals early alterations related to inflammation and metabolic imbalance may already be present even before lipid levels exceed traditional abnormal thresholds. At the same time, excess lipids can promote local inflammatory responses through several mechanisms, including activation of inflammatory pathways such as the toll like receptor 4(TLR4), nuclear factor kappa B (NF-κB) signaling pathway, lipotoxicity, ER stress, and mitochondrial dysfunction. In addition, oxidized low density lipoprotein (ox-LDL) formed through LDL oxidation can be recognized and internalized by receptors such as CD36 and lectin like oxidized low density lipoprotein receptor 1 (LOX-1), thereby promoting foam cell formation and inducing proinflammatory responses (105–109). Overall, lipid concentrations within the upper normal range may contribute to the development and progression of metabolic diseases, including T2DM, metabolic dysfunction associated fatty liver disease, OB, and thyroid dysfunction, through shared mechanisms such as chronic low grade inflammation, oxidative stress, ER stress, and mitochondrial dysfunction, as illustrated in **Figure 2** (39).

During the pathological progression of T2DM, sustained lipid supply drives overload of the mitochondrial electron transport chain, resulting in electron leakage and excessive ROS generation. This oxidative stress, in conjunction with lipid overload, disrupts ER calcium homeostasis and protein-folding capacity, thereby triggering the unfolded protein response. The intracellular crisis arising from mitochondrial dysfunction and ER stress not only directly impairs insulin signaling but also induces systemic low-grade inflammation (110). Inflammation originating from adipose tissue is driven by caloric excess and organelle functional decompensation, leading to adipocyte hypertrophy, hypoxia, and macrophage infiltration, while concurrently activating the secretion of proinflammatory mediators such as tumor necrosis factor-α (TNF-α) and interleukin-6 (IL-6) from adipocytes (111). In this context, interactions exist among ROS accumulation, ER stress, toll like receptor related inflammatory signaling, and activation of the NLRP3 inflammasome, and these processes collectively contribute to the development and progression of pancreatic β cell dysfunction and peripheral IR in T2DM (112, 113).

Chronic low-grade inflammation serves as a central pathological driver in the progression of MAFLD from simple steatosis to NASH and fibrosis (114, 115). Firstly, hypertrophic visceral adipose tissue undergoes hypoxia, macrophage infiltration, and increased secretion of pro-inflammatory adipokines, thereby sustaining systemic low-grade inflammation while exacerbating hepatic lipotoxicity through FFA spillover. Secondly, this systemic inflammatory state activates local hepatic immune cells, including Kupffer cells, neutrophils, and dendritic cells, promoting the activation of signaling pathways such as NF-κB and the NLRP3 inflammasome, which in turn induce hepatocyte injury and amplify inflammatory responses. Concurrently, chronic inflammation and oxidative stress establish a vicious cycle, whereby oxidative stress and lipid peroxidation products not only directly damage hepatocytes but also further stimulate immune cell activation (116). Hepatocellular lipotoxicity itself can provoke ER stress and mitochondrial dysfunction, resulting in the release of damage-associated molecular patterns that, via Kupffer cell–mediated signaling, exacerbate liver injury. Ultimately, sustained inflammatory stimuli mediated by cytokines such as TNF-α, IL-6, and TGF-β drive hepatic stellate cell activation and extracellular matrix deposition, rendering fibrosis the structural consequence of chronic inflammation (117).

During the development and progression of OB, abnormal proliferation and hypertrophy of adipose tissue disrupt the local microenvironment, directly impairing mitochondrial respiratory function and increasing ROS production, thereby triggering inflammatory responses. This inflammation is characterized by extensive immune cell infiltration and a phenotypic shift of macrophages from the anti-inflammatory M2 state to the pro-inflammatory M1 state, markedly enhancing the secretion of pro-inflammatory cytokines (118). Concurrently, hypertrophic adipocytes exhibit functional dysregulation, including increased release of inflammatory mediators such as TNF-α, IL-6, and MCP-1. The adipokine secretion profile is also altered, with abnormally elevated leptin levels and markedly reduced adiponectin, thereby weakening their anti-inflammatory and insulin-sensitizing effects (119, 120). Moreover, ER stress and mitochondrial dysfunction limit the lipid storage capacity of adipocytes, resulting in FFA spillover, which further exacerbates lipotoxic damage and amplifies inflammatory signaling via Toll-like receptor activation (121). Collectively, these pathological processes transform adipose tissue into a source of systemic chronic inflammation, thereby promoting the onset of complications.

The synthesis and secretion of thyroid hormones depend on ER protein folding capacity and mitochondrial energy supply (122). Oxidative stress and ER dysfunction associated with systemic chronic inflammation may impair the function of thyroid follicular cells and interfere with thyroid hormone synthesis (122, 123). At the same time, the accumulation of ROS and inflammatory conditions may also affect peripheral thyroid hormone metabolism, leading to reduced conversion of T4 to triiodothyronine (T3) (124, 125). In addition, oxidative stress and alterations in the inflammatory environment may promote abnormal immune responses against thyroid antigens, thereby contributing to the development of autoimmune TD (126). Inflammatory mediators can also disrupt the hypothalamic–pituitary regulatory axis, suppressing TRH and TSH secretion (123). In summary, organelle dysfunction related to metabolic stress and chronic inflammation may interact with each other and collectively contribute to the development and progression of thyroid dysfunction by affecting multiple processes, including hormone synthesis, peripheral metabolism, central regulation, and the immune environment.

### 3.2 Common molecular signaling pathways

**Figure 3:**
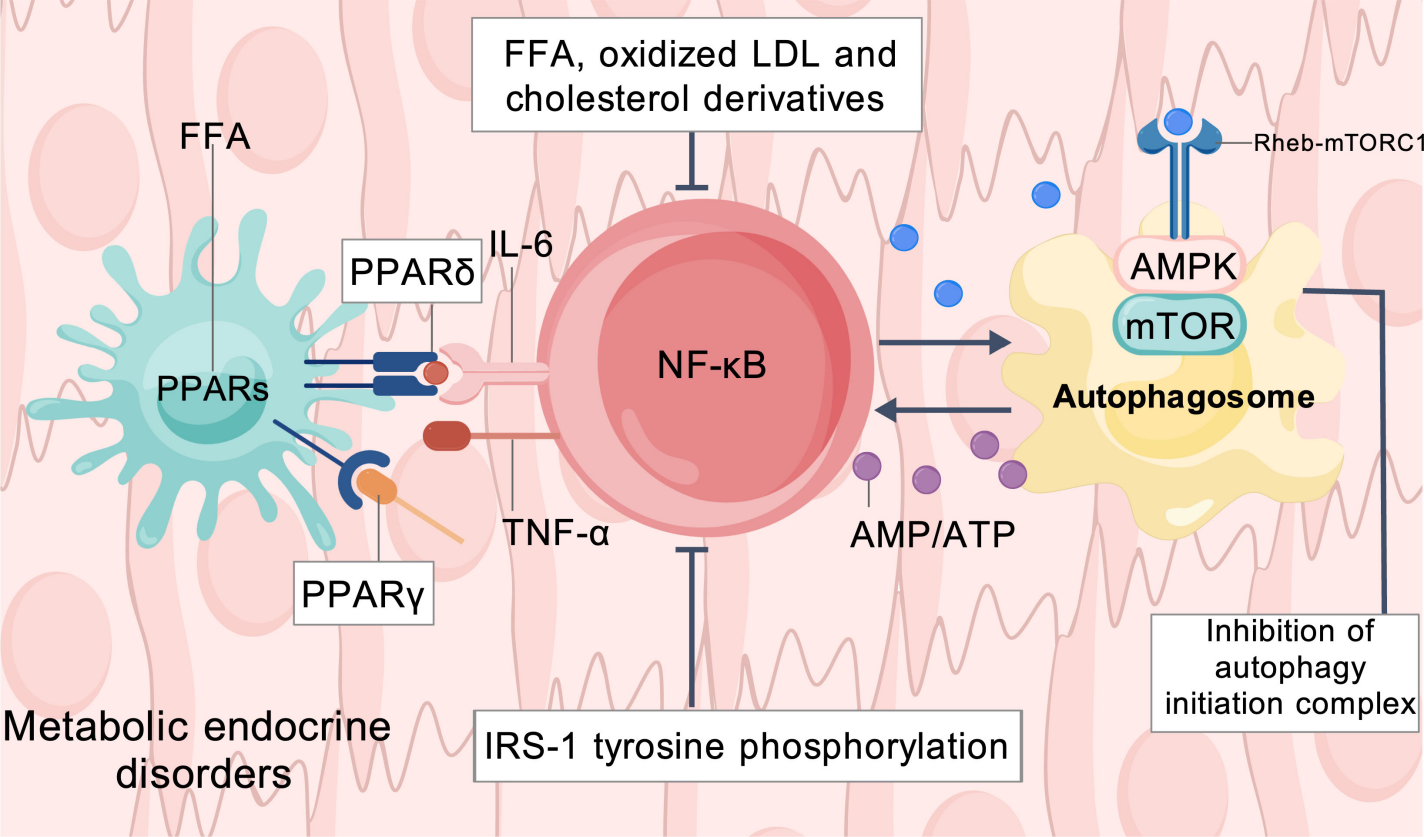
Common molecular signaling pathways.

The pathophysiological effects of dyslipidemia are associated with the dysregulated modulation of multiple key molecular signaling pathway networks. Notably, even within the conventional reference range, subtle variations in lipid levels may still show a continuous association with related pathological processes. Cellular responses to lipids, energy substrates, and their metabolic intermediates are continuous rather than discrete threshold-switch reactions, indicating that subtle variations within the normal lipid range can still exert significant effects on metabolic and endocrine processes. Among these pathways, the AMP-activated protein kinase (AMPK)/mechanistic target of rapamycin (mTOR) signaling axis, the peroxisome proliferator-activated receptor (PPAR) nuclear receptor family, and NF-κB inflammatory pathway serve as shared critical hubs, as shown in **Figure 3** (39).

#### 3.2.1 AMPK/mTOR signaling axis

AMPK functions as a classical cellular energy sensor, becoming activated under energy-deficient conditions, such as an elevated AMP/ATP ratio, to promote catabolic processes. In contrast, the mTOR pathway drives anabolic metabolism and cell growth under nutrient-rich conditions, together constituting a central signaling axis that maintains energy homeostasis (127, 128). Under conditions of chronic nutrient excess, elevated plasma TG and the accumulation of lipid substrates such as FFA often indicate lipid overload and an excess of energy substrates (129, 130). Existing studies suggest that this state is associated with reduced AMPK activity, enhanced lipogenesis, and activation of the mTOR signaling pathway (130, 131). At the same time, nutrient and growth signals can promote anabolic metabolism through mechanisms such as Rheb mediated activation of mTORC1 (132, 133). In addition, mTORC1 can suppress autophagy activity by inhibiting autophagy initiating molecules such as ULK1, thereby affecting the clearance of abnormal proteins and lipid droplets and contributing to increased metabolic stress related to lipid accumulation (134, 135). Imbalance in the AMPK/mTOR signaling axis has been observed in metabolic disorders such as OB, IR, and MAFLD, and is associated with disease progression (127, 136–139). These findings suggest that the lipid overload state reflected by elevated lipid components may contribute to the development of dysregulation in metabolic pathways related to nutrient excess.

#### 3.2.2 Peroxisome proliferator-activated receptors family

PPARs constitute an important family of nuclear receptors involved in the regulation of lipid metabolism, glucose homeostasis, and inflammatory responses (140). Their endogenous ligands include FFA and other lipid derived molecules; therefore, PPAR activity is closely associated with changes in lipid related signaling. Different FAs, as well as variations in their concentrations, can activate PPAR subtypes to varying degrees, thereby influencing fatty acid oxidation, lipid storage, and energy metabolism (141). PPARα primarily promotes fatty acid β oxidation in the liver and skeletal muscle (142). Moreover, a long term increase in lipid burden may be accompanied by dysregulation of PPAR signaling, which may consequently reduce fatty acid oxidation and increase the risk of hepatic lipid accumulation (143). In adipose tissue, PPARγ promotes adipogenesis and lipid storage, thereby helping to maintain adipose tissue metabolic function. However, when the capacity of adipose tissue to handle excess energy declines, FFA can be transported to non-adipose tissues, consequently leading to ectopic fat deposition (144). PPARδ, by contrast, is broadly involved in fatty acid oxidation, energy metabolism, and inflammatory regulation; notably, certain FAs can activate PPARδ at physiologically relevant concentrations (145). Overall, alterations in PPAR signaling are closely associated with diseases such as OB, IR, and MAFLD.

#### 3.2.3 NF-κB signaling pathway

NF-κB, as an important transcription factor in inflammatory responses, is closely associated with chronic low grade inflammation in metabolic diseases. Multiple lipid components, including FFA and oxidized LDL, can act as endogenous danger signals and participate in the activation of NF-κB related inflammatory pathways. Existing experimental studies have shown that FFA such as palmitic acid can promote NF-κB activation through TLR4/MyD88 related mechanisms, with an enhancing trend observed within a certain concentration range. Oxidized LDL can likewise induce NF-κB activation and proinflammatory responses such as TNF α production, and biological effects have been observed even at low doses (146). In addition, lipid accumulation can activate the NLRP3 inflammasome, promote IL 1β maturation, and amplify inflammatory responses together with NF-κB related inflammatory signaling (147). Activation of NF-κB can increase the expression of proinflammatory factors such as TNF α and IL 6 and impair insulin signaling, thereby promoting the development and aggravation of IR (148, 149). These findings suggest that relative increases in lipid related components may not exert biological effects only after reaching clearly abnormal thresholds, but may instead participate in tissue inflammatory responses at an earlier stage. Overall, NF-κB related inflammatory signaling represents one of the key molecular pathways underlying chronic low grade inflammation and is also a shared mechanism associated with metabolic diseases such as T2DM, MAFLD, and OB.

### 3.3 Common cellular effects

**Figure 4:**
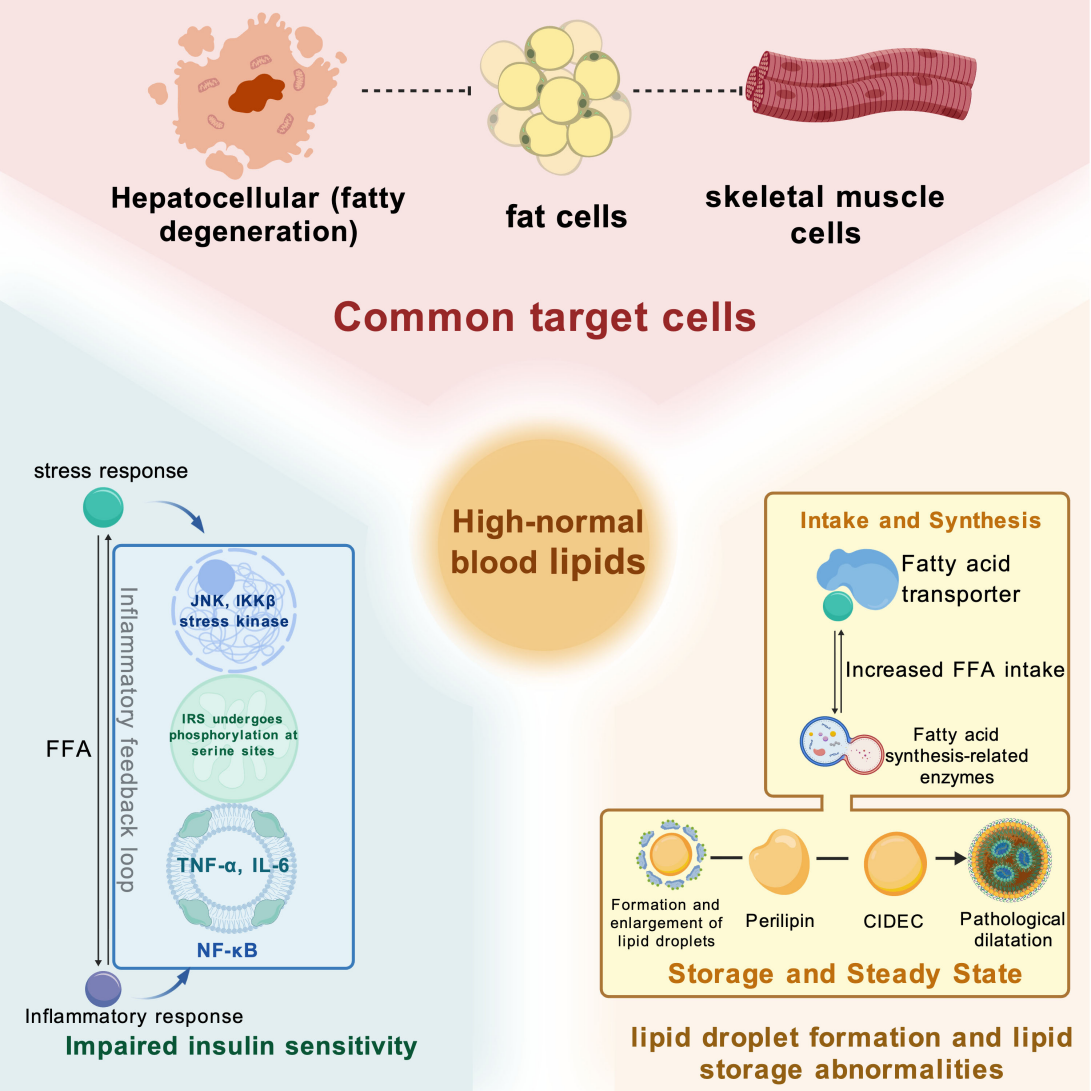
Common cellular effects.

Within the normal lipid range, mild lipid fluctuations, although they do not usually reach the threshold for clinical abnormality, may still elicit biologically meaningful cellular responses in certain tissues and cell types. These responses may be jointly influenced by the aforementioned pathophysiological basis and related molecular signaling pathways and may, in some disease contexts, exhibit a certain degree of dose dependence. Consequently, they may provide a cell biological explanation for the potential links between metabolic and endocrine diseases, as shown in **Figure 4** (39).

#### 3.3.1 Impaired insulin sensitivity

Insulin sensitivity reflects the degree to which the body responds to insulin action and primarily involves the regulation of glucose metabolism, while also being accompanied by modulation of lipid metabolism and related processes. Existing studies have shown that elevated FFAs can dose-dependently impair insulin action even within the physiological range, whereas increases in TG within the normal range are also associated with IR. Taken together, these findings suggest that alterations in lipid metabolism may contribute to early metabolic impairment before overt abnormalities in glucose metabolism become apparent (71, 150, 151).

In adipose tissue, the liver, and skeletal muscle, elevated FFAs, increased TG, and the accumulation of related lipid species are associated with lipotoxic stress and impaired insulin signaling. FFAs can activate stress-related kinases such as JNK and IKKβ, thereby increasing serine phosphorylation of insulin receptor substrate (IRS) and disrupting its normal function. Consequently, PI3K-related insulin metabolic signaling is attenuated, with further effects on the downstream AKT pathway (152–154). In the liver and skeletal muscle, elevated FFAs and the accumulation of lipid intermediates such as diacylglycerol can also activate signaling molecules including protein kinase C, which further suppresses insulin signaling (155).

At the same time, elevated FFAs are associated with a state of low-grade inflammation and may be accompanied by increased levels of inflammatory cytokines such as TNF-α and IL-6. These changes may further exacerbate IRS inhibition and impair insulin signaling through pathways including NF-κB and JNK (154). Based on the available evidence, it can be inferred that, as FFA levels increase, their detrimental effect on insulin sensitivity may progressively intensify. Similarly, other blood lipid components, such as TG, may exert effects in the same direction, although more direct evidence is still needed to confirm this possibility (151, 155). Therefore, elevated FFAs, increased TG, and the accumulation of related lipid species may participate in the shared pathological processes underlying T2DM, IR, and MAFLD.

#### 3.3.2 Lipid droplet formation and abnormal lipid storage

Lipid droplets, as dynamic organelles for the intracellular storage of TG and cholesteryl esters, are tightly regulated to maintain cellular homeostasis. When lipid load increases and this balance is disrupted, cells enter a state of lipid overload, providing the pathological basis for ectopic lipid deposition in OB, MAFLD, and other metabolic diseases.

Elevated blood lipid levels, particularly increased FFAs, can promote the formation and enlargement of lipid droplets in hepatocytes, adipocytes, and skeletal muscle cells, thereby increasing the intracellular burden of lipid storage. FFAs serve as substrates for TG synthesis, while enhanced DNL can further increase TG production; together, these processes promote intracellular lipid accumulation (156, 157). As blood lipid levels rise, the expression or activity of fatty acid transport proteins on the cell membrane may also increase, thereby enhancing cellular FFA uptake and further aggravating the intracellular lipid burden (157, 158).

Lipid droplet-associated proteins, including perilipin and CIDEC, are involved in the stabilization, fusion, and lipolytic regulation of lipid droplets. When the expression of these proteins becomes dysregulated, the efficiency of lipid droplet mobilization and degradation may be reduced, thereby promoting further lipid droplet enlargement and exacerbating intracellular lipid accumulation (156, 158, 159). As the lipid burden increases, lipid droplets may gradually shift from an early adaptive storage state to abnormal expansion, which is associated with pathological lipid accumulation (156, 160).

Abnormal lipid droplet formation and lipid storage constitute central features of hepatocellular steatosis in MAFLD and also provide an important basis for ectopic lipid deposition in non-adipose tissues in metabolic disorders such as T2DM (159, 160). Under different disease contexts, elevated blood lipid levels may progressively increase the lipid burden at both the cellular and tissue levels, thereby further disrupting metabolic homeostasis. Existing studies suggest that this cumulative effect may become more pronounced with increasing exposure levels in certain lipid components and tissues; however, the magnitude of this effect is not entirely consistent across different settings (160, 161).

## 4 Differential dose–response relationships within the normal lipid range and related mechanisms

The dose–response relationships between lipid components and the risk of metabolic and endocrine diseases may exhibit a certain degree of disease specificity and nonlinear characteristics. This heterogeneity is related not only to variations in lipid levels, but may also be jointly influenced by individual metabolic background, energy status, and endocrine homeostasis. The risk patterns, key features, and related mechanisms of different lipid components in specific disease contexts are summarized below (**Table 3**).

**Table 3:** Dose–response patterns of major lipid components across metabolic diseases.

Lipid component	Disease / phenotype	Dose–response pattern	Key interpretation
LDL-C	T2DM	Heterogeneous	Inverse for risk; nonlinear for related phenotypes
	MAFLD	Phenotype-dependent	Related to adiposity and metabolic status
	OB	Phenotype-dependent/ nonlinear	May vary with body weight status and fat distribution
	TD	Positive linear	Hormone-regulated lipid alteration
HDL-C	DM	U-shaped / nonlinear	Function may outweigh quantity
	MAFLD	Association observed; shape uncertain	Low HDL-C and HDL dysfunction are linked to disease risk
	OB	Function-related alteration	Changes in HDL metabolism, composition, and subclasses
	TD	No consistent pattern	Variable hormonal influence
TG	DM / T2DM risk	Threshold / U-shaped / nonlinear	Modified by glycemic status and IR
	MAFLD	Positive, partly nonlinear	Stronger signal in composite indices
	OB	Nonlinear, with variable thresholds	Thresholds and curve shape vary by population and metabolic status
	TD	Positive association with TSH	Observed even within the reference range
TC	MAFLD	Positive association	Linked to hepatic lipid burden
	T2DM	Dynamic positive association	Rising TC trajectory is associated with higher CVD risk
	OB	Complex interactive pattern	Influenced by diet, rhythm, and sex
	TD	Bidirectional hormone-related change	Varies with thyroid functional state

### 4.1 Dose–response characteristics of LDL-C in different diseases

The dose–response relationship of LDL-C across different diseases shows a certain degree of heterogeneity and cannot always be adequately explained by the simple linear assumption that “the lower, the better.” In T2DM, large-scale genetic cohort studies have suggested a stable dose–response relationship between genetically determined LDL-C levels and the risk of T2DM, with lower genetically predicted LDL-C levels being associated with a higher risk of T2DM (162). In addition, a cross-sectional study reported an inverted U-shaped relationship between LDL-C levels and cognitive function in patients with T2DM (163). Furthermore, another cross-sectional study showed an inverted U-shaped relationship between BMI and LDL-C in male patients with T2DM, suggesting that body weight status may influence the variation pattern of LDL-C (164).

In populations with MAFLD, the dose–response relationship between LDL-C and hepatic fat content exhibits clear phenotypic heterogeneity. In overweight or obese MAFLD, hepatic fat content shows a dose–response relationship with lipid indicators such as LDL-C, whereas this relationship is not entirely consistent across other phenotypes (165). This pattern may be related to lipid metabolism disorders and altered lipid distribution under conditions of severe OB; however, the precise mechanisms remain to be further elucidated. These nonlinear features suggest that the association between LDL-C and related metabolic outcomes may be influenced by the degree of fat deposition and systemic metabolic status. In studies related to thyroid function, elevated TSH has shown a linear dose–response relationship with increased LDL-C levels, whereas patients with hypothyroidism generally exhibit higher LDL-C levels than controls, reflecting the direct regulatory effect of endocrine hormones on blood lipid levels (95, 166).

Overall, the dose–response relationship of LDL-C varies across different disease contexts and may manifest as a linear association, an inverse association, or a nonlinear pattern. These findings indicate that the assessment of LDL-C-related risk cannot be separated from the specific disease state, metabolic background, and study endpoint. Instead, it should be interpreted in conjunction with the degree of fat deposition, body weight status, and the endocrine milieu.

### 4.2 Dose–response characteristics of HDL-C in different diseases

HDL-C is one of the blood lipid components that most characteristically exhibits a U-shaped dose–response curve, and its functional quality may be more important than its absolute quantity in the assessment of metabolic disease risk. In patients with DM, HDL-C may show a significant U-shaped association with clinical outcomes. In addition, another study reported that HDL-C was generally negatively correlated with HbA1c in patients with DM, although a nonlinear relationship with an inflection point at approximately 60 mg/dL was observed in certain subgroups (167, 168). These findings suggest that, when HDL-C reaches relatively high levels, the risks of cardiovascular mortality and all-cause mortality may paradoxically increase, thereby further supporting a nonlinear relationship between HDL-C and adverse outcomes in the context of diabetes. At the same time, these observations also indicate that high HDL-C levels do not necessarily signify intact anti-inflammatory activity and reverse cholesterol transport capacity, but may instead reflect a state of functional impairment. Under conditions of diabetes and metabolic dysregulation, HDL subclasses and their anti-inflammatory properties may also be altered, which further suggests that the functional status of HDL cannot be represented solely by HDL-C concentration.

Existing studies suggest that persistently low HDL-C levels are associated with incident MAFLD, whereas HDL dysfunction is also related to the onset and progression of NAFLD/MAFLD. However, whether HDL-C shows a linear or approximately linear relationship with hepatic fat content remains to be further clarified. This association may also differ across body weight phenotypes; nevertheless, consistent evidence is still lacking as to whether non-obese MAFLD is more sensitive to reductions in HDL-C (169). In OB, the dose–response characteristics of HDL-C may no longer be reflected solely by conventional changes in concentration, but may instead be manifested more prominently through alterations in HDL metabolism, composition, and subclass distribution (170). In TD, HDL-C is also subject to hormonal regulation; however, compared with LDL-C, both the magnitude and direction of its change are less consistent, and it may present as a slight increase, a decrease, or no significant change. Therefore, its nonlinear features remain unclear at present (171).

Traditionally, HDL-C has been regarded as a protective factor. However, current evidence suggests that extremely high circulating HDL-C levels do not necessarily translate into sustained metabolic benefit and may, in some cases, even be associated with adverse outcomes. This further indicates that reliance on HDL-C concentration alone is insufficient to fully capture its biological role (172).

### 4.3 Dose–response characteristics of TG in different diseases

TG is a sensitive risk signal in most metabolic diseases, and its dose–response relationship may display a certain directional pattern while also being substantially modified by glycemic and metabolic status.

In the prediction of DM risk, the dose–response relationship of TG may exhibit a clear threshold-like structure. For example, in a cohort study of Japanese men with normal glucose levels and concomitant NAFLD, the association between TG and the risk of incident T2DM followed a U-shaped dose–response pattern, with an inflection point at approximately 53 mg/dL (173). Above this threshold, the risk increased progressively with rising TG levels. In addition, the TyG index, which is regarded as an indicator related to IR, has also shown a U-shaped dose–response relationship in the prediction of diabetes, although the inflection point may vary across different metabolic backgrounds (174, 175).

In MAFLD, TG and related composite indicators are generally associated with an increased risk of disease. Compared with TG alone, composite indices such as the TyG index integrate information on both glucose and lipid metabolism and may therefore demonstrate more pronounced nonlinear dose–response characteristics in certain populations. At the same time, indicators such as the TG/HDL-C ratio have also been reported to show a persistent positive association with the development of MAFLD (176–178).

In the context of OB, the relationship between TG-related risk indicators and clinical outcomes may also follow a nonlinear pattern; however, the specific thresholds and curve shapes remain influenced by the study population and metabolic background (179). This may be related to enhanced lipolysis, increased fatty acid influx, and abnormalities in re-esterification and TG synthesis under obese conditions, although their precise effects on the shape of the risk curve still require further investigation (180, 181).

TG levels are also associated with thyroid status. Even among individuals with TSH levels within the reference range, TG may increase in parallel with rising TSH, suggesting that its dose–response relationship is generally positively directed (95).

Overall, the dose–response characteristics of TG are not fixed and may manifest as a continuous positive association, a threshold effect, or a nonlinear pattern. These findings suggest that TG is not only a marker of disordered lipid metabolism, but also an important signal linking impaired glucose metabolism, fatty liver formation, and systemic metabolic imbalance.

### 4.4 Dose–response characteristics of TC in different diseases

The association between TC and metabolic diseases does not follow a single linear pattern; rather, it shows a certain degree of disease specificity and population heterogeneity. Its dose–response relationship ranges from positive associations to nonlinear interactions, and this complexity may reflect differences in the body’s capacity to regulate lipid metabolism under distinct pathophysiological conditions.

In MAFLD, the dose–response relationship of TC generally exhibits a relatively stable positive association. Cohort studies have shown that, with increasing quartiles of TC, TG, and LDL-C, the risk of fatty liver disease rises significantly (178). This relationship supports the role of TC as an important metabolic factor in this disease and further suggests that, during the development of hepatic steatosis, sustained accumulation of circulating TC may contribute to the pathological progression from simple steatosis to NASH by inducing ER stress and inflammatory responses (182–184).

In contrast to MAFLD, the dose–response characteristics of TC in T2DM may reflect a more complex pattern, shifting from a relatively simple linear association to multifactorial interactive effects. On the one hand, TC shows a clear risk-related trajectory during disease progression. Dynamic cohort studies have confirmed that, in patients with T2DM, changes in TC levels from lower to higher levels before and after diagnosis are significantly positively associated with CVD risk, suggesting that long-term exposure to elevated cholesterol may contribute to the increased cardiovascular risk associated with T2DM (185). On the other hand, under the background of OB, disruption of TC homeostasis appears to involve complex interactive effects. Studies have shown that TC levels are not regulated solely by lipid burden in a unidirectional manner, but are instead jointly modified by exogenous dietary load, biological rhythms, and glycemic homeostasis (186). Such interactive features may also vary by sex. In particular, the distribution pattern of TC in female patients may differ from that in male patients, while sex hormones, especially estrogen, play regulatory roles in lipid metabolism and cholesterol transport (187). Therefore, the evaluation of TC in patients with T2DM and OB should no longer be confined to the linear logic of static concentrations, but should instead move toward a dynamic, multidimensional, interactive, and sex-specific framework for comprehensive management.

In TD, thyroid functional status exerts a significant influence on TC levels and is characterized by hormone-regulated changes. In hypothyroidism, reduced LDL receptor activity slows TC clearance, leading to pathological elevation; in contrast, hyperthyroidism may accelerate cholesterol turnover and thereby result in abnormally reduced TC levels (95, 166, 188). These endocrine axis-mediated fluctuations in lipids indicate that TC is not only a marker associated with metabolic risk, but also, to some extent, a reflection of changes in the body’s metabolic state.

## 5 Metabolic health and personalized lipid management

### 5.1 Assessment of metabolic health and application of biomarkers

A single biomarker is insufficient to capture the complexity of metabolic dysregulation, necessitating the integration of multi-omics approaches. Lipidomics, leveraging high-resolution mass spectrometry, overcomes the limitations of conventional assays by characterizing specific lipid subtypes—such as ceramides and plasmalogens—thereby elucidating their critical roles in IR and cardiovascular pathology and providing novel targets for early diagnosis. Genomic approaches, including genome-wide association studies and Mendelian randomization, further clarify the shared genetic underpinnings between lipid traits and metabolic syndrome. Integrating polygenic risk scores with lipidomic and phenotypic data substantially enhances risk stratification and informs precision prevention strategies (189, 190). Moreover, machine learning algorithms enable the extraction of preclinical signals that are often overlooked by conventional testing from high-dimensional omics datasets, facilitating the identification of high-value biomarkers and providing a robust foundation for individualized intervention (191).

### 5.2 Personalized lipid management and intervention strategies

Personalized lipid management should integrate lifestyle modification, nutritional intervention, and pharmacotherapy. Lifestyle interventions—including adherence to a balanced diet, regular physical activity, and effective weight management—have been demonstrated to improve LDL-C, HDL-C, TG, and small dense LDL levels, thereby mitigating the risk of metabolic diseases (192–195). Functional foods and dietary supplements, such as plant sterols and omega-3 FAs, can further optimize lipid profiles (196). In addition, pharmacological interventions are essential for individuals at high risk or when lifestyle measures alone are insufficient. Therapeutic options include statins, ezetimibe, PCSK9 inhibitors, fibrates, and high-dose omega-3 FAs. Moreover, emerging targeted therapies and certain antidiabetic or anti-OB agents—such as GLP-1 receptor agonists, SGLT2 inhibitors, and pioglitazone—offer dual benefits by improving both lipid profiles and MAFLD, thereby achieving comprehensive metabolic and cardiovascular protection (197, 198). Effective lipid management requires an integrated understanding of systemic metabolism, accounting for glucose homeostasis, insulin sensitivity, and inflammatory status, and must be precisely tailored to the individual’s genetic background and potential drug-related risks.

### 5.3 Limitations and future research directions

Although this review systematically elucidates the significant dose–response relationships between different lipid components and metabolic diseases, substantial challenges remain with respect to clinical translation. First, the current body of evidence is characterized by marked heterogeneity. Most included studies are observational cohorts, and differences in baseline population characteristics, such as ethnicity, age, and metabolic status, as well as in the standardization of lipid measurements and disease diagnostic criteria, limit the generalizability of the conclusions. More importantly, although this review focuses on dose–response relationships within the conventionally normal lipid range, the currently available evidence is not uniformly restricted to populations with entirely normal lipid levels. Therefore, across multiple disease contexts, the observed associations should be interpreted as suggesting the possible presence of latent risk gradients even within the normal range. Second, causal inference and mechanistic elucidation remain incomplete. Although existing studies have identified associations between lipid fluctuations and disease risk, molecular validation in humans remains insufficient regarding how abnormal changes in different lipid components may induce tissue and organ injury through specific signaling pathways. Consequently, clinical decision-making remains difficult to shift from statistical risk prediction toward pathology-based targeted intervention.

To address the issues outlined above, future research should prioritize the establishment of a high-dimensional precision identification framework. First, dedicated studies are urgently needed in individuals strictly defined as being within the normal lipid range in order to determine whether the observed dose–response patterns truly reflect risk gradients operating within the conventionally defined normal lipid spectrum. To reduce interstudy heterogeneity, large-scale, multicenter, and international cohort data should be integrated, and advanced analytical strategies, including artificial intelligence and machine learning, should be applied to control for potential confounding factors and identify individualized risk indicators. To overcome the current limitations of mechanistic research, high-throughput lipidomics and spatial transcriptomics may be employed to systematically characterize the dynamic effects of lipid fluctuations on metabolic networks. At the same time, assessment strategies should shift from static measurements toward dynamic monitoring, and emerging monitoring technologies may also be considered for longitudinal assessment of metabolic fluctuations, thereby enabling a more precise evaluation of the long-term effects of cumulative lipid burden. Finally, long-term prospective cohort studies and Mendelian randomization analyses would help strengthen the reliability of causal inference regarding dose–response relationships. By integrating multidimensional data, including genetic susceptibility, environmental exposures, and lifestyle factors, future studies may help promote a transition in lipid management strategies from standardized intervention models toward more individualized approaches to prevention and risk stratification, thereby providing stronger evidence-based support for the optimization of metabolic health management.

## 6 Conclusion

Within the conventionally defined “normal” range, the dose–response relationships of different lipid components in metabolic and endocrine diseases may display a certain degree of specificity, and these patterns may be jointly influenced by shared pathophysiological processes, such as inflammation and oxidative stress, as well as by tissue-specific regulatory mechanisms. However, the available evidence remains heterogeneous, and the strength of evidence is not uniform across different lipid components and disease contexts within the “normal” range. These findings suggest that traditional reference ranges may not be sufficient to fully capture the complex associations between lipid profiles and health risk. Therefore, future studies should systematically take individual physiological differences into account and further delineate background-dependent risk patterns within the normal lipid range, thereby promoting more precise individualized lipid management and optimizing risk prevention for metabolic diseases. 
